# Account of an Endemic Disease in Norway, Called Radesyge

**Published:** 1801-07

**Authors:** 


### *Account of an Endemic Disease in Norway, called* RADESYGE.

ANY bad or foul diſeaſe is called by the common people in Norway, *Radesyge* (from Rade, bad, foul) but the diſeaſe which is particularly meant by this name, was thought by ſome phyſicians to originate from the Oriental leproſy, or elephantiaſis, and to have been tranſplanted into Norway in the times of the cruſades. This conjecture, however, ſeems not to be well grounded, though the diſeaſe ſometimes approaches in its ſymptoms to that evil. It is rather probable that it has a venereal origin, origin, as appears from the defcription Dr. Munk gives of it, who had frequently an opportunity of making feveral obferva-tions on its nature, and the method of curing it.

"The difeafe begins fometimes with topical complaints, chancres, buboes, gonorrhœas, &c. but moft frequently it comes on with wandering pains, increafing during the night, which are followed by ulcers in the mouth, attacking by degrees the *uvula*, the *velum palatinum pendulum*, and the palate bones; at the fame time piles appear round the anus, and ulcers on the body, particularly about the fhoulders and the belly, which have the appearance of venereal ulcers, but are frequently cover-ed with a thick cruft. In fome cafes excrefcences come on inftead of the ulcers. The Radefyge differs particularly from the lues, by appearing without any previous topical complaints. Sometimes the difeafe proceeds fo flowly, that the patient may walk about for feveral months without fuffering much incon-venience; but in other cafes its progrefs is very rapid. No-thing certain can be faid with refpect to the manner of infec-tion and propagation, as moft patients are ignorant whence they obtain it, though others have apparently contracted it by a concubitus impurus. It is eafily propagated, as the common people in Norway fleep together naked under woollen cover-ings, or in the mountainous parts on fkins, the hairy fide of which is turned towards the naked body; and a principal caufe of its propagation is the extreme uncleanlinefs that prevails there among the lower ranks of people.

"The beft remedies againft that difeafe, are decoctions of Lignum guaiacum, farfaparilla, &c. and principally mercurials, given till they bring on a copious falivation. No particular regimen is to be obferved.

"Exanthemata of the very worft kind, accompanied with knobs in the fkin, infupportable itching, offenfive fmell, falling off of the eye-brows, and other fymptoms, which impart to this difeafe the character of leprofy, or elephantiafis, are fome-times obferved in that country; at which no perfon will be furprized when he confiders that a great part of its inhabitants are frequently on the fea in cold and wet feafons, drink a large quantity of brandy, and live upon putrid and ftinking fifh, to the exhalation of which they are continually expofed.

Pharmaceutical

